# Nrf2/HO-1 signaling in retinal neurodegenerative diseases: redox homeostasis, cytoprotection, and therapeutic perspectives

**DOI:** 10.3389/fmed.2026.1910230

**Published:** 2026-09-21

**Authors:** Yu Tang, Xiaolei Yao, Qinghua Peng

**Affiliations:** 1 The First Clinical College of Chinese Medicine, Hunan University of Chinese Medicine, Changsha, Hunan, China; 2 Department of Biochemistry & Molecular Biology, Faculty of Medicine, University of British Columbia, Vancouver, BC, Canada; 3 Hunan Provincial Key Laboratory for Prevention and Treatment of Ophthalmology and Otolaryngology Diseases with Chinese Medicine, Changsha, Hunan, China

**Keywords:** HO-1, neuroprotection, Nrf2, redox homeostasis, retinal neurodegenerative diseases

## Abstract

Retinal neurodegenerative diseases (RNDs) represent a major group of disorders leading to irreversible visual impairment. Persistent oxidative stress, mitochondrial dysfunction, inflammatory responses, lipid peroxidation, and disruption of neurovascular unit homeostasis are closely implicated in retinal neuronal and vascular injury. Nuclear factor erythroid 2–related factor 2 (Nrf2) and its downstream effector heme oxygenase-1 (HO-1) constitute a key regulator*y* axis in cellular antioxidant defense and adaptive stress responses. Current preclinical evidence suggests that Nrf2/HO-1 signaling may participate in retinal cytoprotection, inflammatory regulation, and maintenance of neurovascular homeostasis in RNDs by modulating redox homeostasis and interconnected injury responses. This review critically synthesizes the cytoprotective roles, evidence boundaries, translational challenges, and future directions of Nrf2/HO-1 signaling in RNDs, organized around retinal cell types, redox homeostasis-related injury mechanisms, disease-specific evidence, and intervention strategies. Further translation will require stronger causal validation, improved ocular delivery, and better cell type-specific targeting.

## 1 Introduction

According to estimates from the Global Burden of Disease Study, approximately 43 million people worldwide were blind in 2020, and another 295 million had moderate to severe vision impairment (1). Retinal neurodegenerative diseases (RNDs) constitute an important group of disorders leading to irreversible visual impairment (2). They are characterized by disease-specific patterns of progressive retinal cell loss and neural injury, including photoreceptor degeneration with associated retinal pigment epithelium (RPE) dysfunction or degeneration in age-related macular degeneration (AMD) and retinitis pigmentosa (RP), endothelial cell damage in diabetic retinopathy (DR), and retinal ganglion cell (RGC) loss with optic nerve axonal injury in glaucoma (2, 3). Although these diseases differ in etiology, affected cell types, and clinical manifestations, oxidative stress, mitochondrial dysfunction, hypoxia-related signaling, inflammatory responses, and disruption of neurovascular unit (NVU) homeostasis have been repeatedly observed across multiple retinal degenerative disorders and are considered key pathological processes contributing to the progression of RNDs (3–6).

Nuclear factor erythroid 2–related factor 2 (Nrf2) is a key transcriptional regulator of cellular responses to oxidative and electrophilic stress. By binding to antioxidant response elements (AREs), Nrf2 induces the expression of multiple genes involved in antioxidant defense and detoxification (7, 8). Its downstream regulatory network includes heme oxygenase-1 (HO-1), NAD(P)H quinone oxidoreductase 1 (NQO1), enzymes involved in glutathione synthesis and metabolism, and glutathione S-transferases (GSTs). Together, these effector molecules contribute to reactive oxygen species (ROS) and reactive nitrogen species (RNS) clearance, maintenance of cellular redox homeostasis, and stress-adaptive responses (7, 8). Rather than functioning as a direct stoichiometric ROS scavenger, Nrf2 acts as an upstream transcriptional regulator of endogenous redox-adaptive programs (7, 8). Beyond its canonical antioxidant functions, studies in the nervous system and in neural injury contexts suggest that Nrf2/HO-1 signaling may also be involved in mitochondrial homeostasis, regulation of inflammatory responses, and processes related to lipid peroxidation and ferroptosis (8–11). Given the intertwined pathological background of oxidative stress, metabolic dysfunction, and inflammation in RNDs, Nrf2/HO-1 signaling may represent an important regulatory node linking redox homeostasis to cytoprotective responses.

In recent years, accumulating preclinical studies have examined Nrf2/HO-1-associated signaling in specific retinal injury contexts. Representative studies in RGC/optic nerve injury, diabetic retinal microvascular injury, and RPE oxidative injury models have linked Nrf2/HO-1-related responses to attenuation of oxidative stress, inflammation, ferroptosis-related changes, or cell-death phenotypes (12–14). These findings suggest that Nrf2/HO-1 may participate in adaptive protective responses across different retinal cell types, including RGCs, retinal microvascular endothelial cells, and RPE cells, under pathological stress conditions. However, current evidence remains largely preclinical and intervention-specific, with substantial heterogeneity in disease context, retinal cell type, injury paradigm, and depth of pathway validation. Further synthesis across cell types, disease settings, and pathological stress networks is therefore needed to clarify the role of Nrf2/HO-1 in RNDs.

On this basis, this review first outlines the molecular basis of the Nrf2/HO-1 signaling axis and then critically synthesizes its potential protective roles, evidence boundaries, and translational challenges in RNDs, with an emphasis on retinal cell-type specificity, redox-related injury mechanisms, disease-specific evidence, and intervention strategies.

## 2 Molecular basis of the Nrf2/HO-1 axis

### 2.1 Core components: Nrf2, Keap1, and HO-1

The Kelch-like ECH-associated protein 1 (Keap1)–Nrf2–HO-1 module is centered on the transcription factor Nrf2, its cytoplasmic repressor Keap1, and the downstream effector enzyme HO-1. Nrf2 is a member of the Cap’n’Collar subfamily of basic leucine zipper (bZIP) transcription factors and contains seven conserved Nrf2-ECH homology (Neh) domains, Neh1–Neh7 (15). Its key functional regions include the Neh2 domain, which mediates Keap1 binding, and the Neh1 domain, which is involved in DNA binding and heterodimerization with small Maf (sMaf) proteins (15, 16). Keap1 is a cysteine-rich negative regulator of Nrf2. Its broad complex, tramtrack, and bric-à-brac (BTB) domain mediates homodimerization and binding to the Cullin 3 (Cul3)–RING-box protein 1 (Rbx1) E3 ubiquitin ligase complex, whereas the DGR/Kelch domain recognizes the DLG and ETGE motifs within the Neh2 region of Nrf2. Key reactive cysteine residues in Keap1, including Cys151, Cys273, and Cys288, act as sensors of oxidative and electrophilic stress (15, 16).

### 2.2 Canonical activation and downstream target regulation

Activation of Nrf2 and induction of downstream HO-1 expression can be triggered by various oxidative or electrophilic stimuli, including ROS/RNS-related stress signals, endogenous metabolites, lipid peroxidation products, and natural or synthetic Nrf2 activators (17, 18). In the canonical model, these stimuli primarily oxidize or covalently modify reactive cysteine residues in Keap1, thereby altering Keap1 conformation or weakening its substrate-adaptor function. This impairs Keap1–Cul3–Rbx1-mediated ubiquitination and degradation of Nrf2, allowing Nrf2 to accumulate, translocate into the nucleus, and initiate an ARE-driven antioxidant and cytoprotective transcriptional program (18). In addition, Nrf2 activity can be modulated by several complementary mechanisms, including Keap1-independent degradation mediated by β-transducin repeat-containing protein, transcriptional coactivation involving CREB-binding protein (CBP) and p300, and phosphorylation-dependent regulation (19).

After stabilization and nuclear translocation, Nrf2 forms heterodimers with sMaf proteins and binds to AREs in the regulatory regions of target genes. This induces the expression of a range of cytoprotective genes involved in antioxidant defense, detoxification of electrophiles and reactive metabolites, and glutathione synthesis and regeneration. Representative targets include HO-1, NQO1, glutamate-cysteine ligase catalytic subunit (GCLC), and glutamate-cysteine ligase modifier subunit (GCLM) (20–22). Functionally, Nrf2 downstream targets and effector networks can be broadly grouped into several categories: antioxidant and detoxifying enzymes, such as HO-1, NQO1, and GSTs, which participate in heme metabolism, quinone reduction, and electrophile detoxification; glutathione synthesis and regeneration-related molecules, including GCLC, GCLM, glutathione reductase, and cysteine transport-associated proteins, which support reduced glutathione (GSH) synthesis and regeneration, thereby helping maintain glutathione-dependent redox homeostasis; iron homeostasis-related proteins, such as ferritin light and heavy chains, which contribute to labile iron sequestration; and the thioredoxin/peroxiredoxin system and other antioxidant enzymes, including thioredoxin (TRX), TRX reductase, peroxiredoxins (PRX), and superoxide dismutase (SOD), which collectively participate in ROS clearance, protein thiol reduction, and maintenance of cellular redox homeostasis (23–25). Among these Nrf2-regulated targets, HO-1 is widely regarded as a representative effector molecule of the Nrf2 signaling axis. HO-1, encoded by the *HMOX1* gene, catalyzes the rate-limiting step of heme degradation, generating carbon monoxide (CO), biliverdin, and ferrous iron (Fe^2+^). Biliverdin can be further converted into bilirubin by biliverdin reductase (16). Under conditions of moderate activation, HO-1-mediated heme catabolism is generally cytoprotective: CO contributes to anti-inflammatory and pro-survival signaling, biliverdin/bilirubin has antioxidant activity, and Fe^2+^ released during the HO-1 reaction is often accompanied by ferritin upregulation, thereby promoting labile iron sequestration and helping limit iron-dependent oxidative injury (16, 26).

### 2.3 Signaling crosstalk and neuroprotective implications of the Nrf2/HO-1 pathway

The Nrf2/HO-1 signaling axis does not operate in isolation. Rather, it is functionally interconnected with stress-sensing pathways such as nuclear factor-κB (NF-κB), hypoxia-inducible factor-1α (HIF-1α), and mitogen-activated protein kinase (MAPK) signaling, collectively contributing to cellular adaptive regulation in response to oxidative stress, inflammatory responses, and hypoxia-related stimuli.

Upon activation, Nrf2 can reduce ROS burden by inducing the expression of HO-1, NQO1, and enzymes involved in glutathione metabolism; in some inflammatory or injury models, this response is accompanied by downregulation of NF-κB-related pro-inflammatory signaling (27). Some studies further suggest that NF-κB/p65 may negatively regulate Nrf2/ARE-dependent transcriptional activity by competing for coactivators such as CBP or by promoting histone deacetylase (HDAC) 3-associated transcriptional repression (28).

Evidence from tumor and non-retinal hypoxia models suggests that context-dependent crosstalk may exist between Nrf2 and HIF-1α. Nrf2 target genes such as NQO1 and TRX1, as well as CO derived from HO-1 activity, may promote HIF-1α accumulation or stabilization, whereas HIF-1α contributes to the expression of genes involved in glycolysis and hypoxic metabolic adaptation (29, 30).

In addition, MAPK signaling can act as an upstream modulator of Nrf2 activity. In a context-dependent manner, activation of extracellular signal-regulated kinase (ERK), p38 MAPK, or c-Jun N-terminal kinase (JNK) may influence Nrf2 stability, nuclear translocation, or transcriptional activity, thereby modulating downstream effectors such as HO-1 and NQO1 (31). MAPK signaling also intersects with NF-κB and phosphatidylinositol 3-kinase/protein kinase B (PI3K/Akt) pathways and participates in the regulation of inflammatory responses, cell-survival/death balance, and redox responses (9). Beyond kinase-dependent regulation, Nrf2 also intersects with autophagy and protein quality-control networks, potentially contributing to the clearance of protein aggregates or oxidatively damaged proteins in neural injury contexts (15, 16).

Therefore, in neural injury settings, the Nrf2/HO-1 pathway should not be viewed merely as a single antioxidant response, but rather as a stress-adaptive network linking redox homeostasis, inflammatory regulation, cell survival, and proteostasis maintenance. The canonical Nrf2/HO-1 signaling framework relevant to RNDs is summarized in Figure 1.

**Figure 1:**
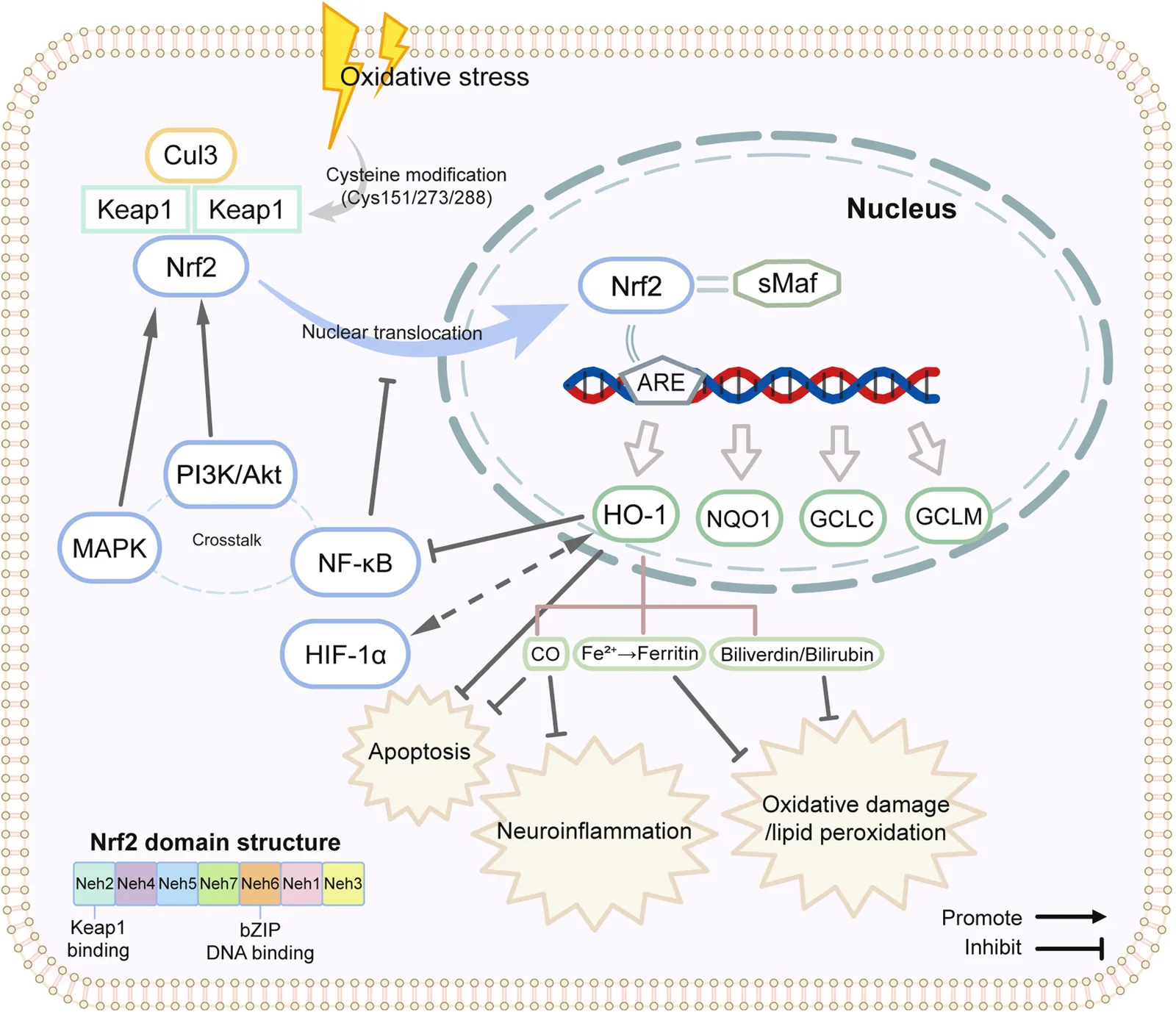
Canonical activation of the Nrf2/HO-1 axis and its crosstalk with stress-response pathways relevant to RNDs. Oxidative stress modifies redox-sensitive cysteine residues in Keap1, reducing Keap1-mediated repression of Nrf2 and enabling Nrf2 nuclear translocation. Nuclear Nrf2 heterodimerizes with sMaf and binds antioxidant response elements (AREs), inducing HO-1, NQO1, GCLC, GCLM, and other cytoprotective genes. HO-1-derived carbon monoxide (CO), biliverdin/bilirubin, and ferritin-associated iron sequestration contribute to the regulation of apoptosis, neuroinflammation, and oxidative damage/lipid peroxidation. The Nrf2/HO-1 axis also intersects with MAPK, PI3K/Akt, NF-κB, and HIF-1α signaling. Arrows and blunt-ended lines indicate promoting and inhibitory relationships, respectively.

## 3 Cell type-associated roles of Nrf2/HO-1 signaling in RNDs

### 3.1 Retinal ganglion cells

RGCs have high energy demands and rely on mitochondrial homeostasis to sustain axonal transport and electrophysiological activity. They are therefore vulnerable to oxidative injury under conditions such as elevated intraocular pressure (IOP), optic nerve injury, and high glucose (HG) stress (32–35). In models of chronic ocular hypertension (OHT), optic nerve crush, and HG-related injury, enhancement of Nrf2/HO-1 or Nrf2-related signaling is frequently associated with reduced oxidative damage, suppression of apoptosis, and improved RGC survival or function (33–36). In selected injury contexts, RGC protection is also accompanied by inflammatory or mitochondrial stress modulation, including inflammasome-related changes in retinal I/R injury (37) and reduced ROS/cytokine responses with preservation of mitochondrial membrane potential in HG-stressed RGC-5 cells (38). Further intervention studies have shown that Nrf2 interference, HO-1 inhibition, or downregulation of upstream regulators such as Parkinson disease protein 7 (PARK7) and Brahma-related gene 1 (BRG1) weakens RGC-protective responses or aggravates RGC injury in corresponding models, supporting the involvement of the Nrf2/HO-1 axis in specific aspects of RGC stress defense (34, 39–41).

### 3.2 Photoreceptors

Photoreceptors are highly susceptible to oxidative injury because the outer retina is subjected to chronic photic stress and high oxygen consumption, while photoreceptor outer segments are rich in membrane lipids that are prone to peroxidation (42). In light-induced injury and H_2_O_2_-treated 661W photoreceptor-like cell models, oxidative stress has been linked to high-mobility group box (HMGB) 2-associated redox-inflammatory signaling and photoreceptor death (43). In rd10 mice, Nrf2/HO-1-related responses are associated with outer nuclear layer (ONL) preservation, improved electroretinographic (ERG) responses, and attenuation of oxidative damage; in H_2_O_2_-injured 661W cells, they are associated with reduced oxidative injury and cell death, suggesting that this axis may participate in antioxidant protection of photoreceptors (43–45).

### 3.3 Retinal pigment epithelial cells

RPE cells form a metabolically active monolayer between the neural retina and the choroid, supporting photoreceptor maintenance, transport functions, redox balance, and outer blood–retinal barrier integrity. Their high metabolic activity and chronic photo-oxidative burden make them particularly vulnerable to oxidative stress, mitochondrial dysfunction, and stress-related morphological or cytoskeletal alterations (46). Current RPE-related evidence for Nrf2/HO-1 involvement is largely derived from *in vitro* studies using the human RPE cell line ARPE-19. In a rotenone-induced oxidative stress model, HDAC inhibitors reduced ROS production, promoted mitochondrial fusion, improved cellular morphology and survival, and activated Nrf2-related antioxidant responses, including HO-1 induction (46). Bisphenol A exposure induced ROS/malondialdehyde (MDA) elevation, reduced SOD/catalase (CAT) and Nrf2/HO-1 expression, and was accompanied by activation of mitochondria-dependent apoptotic pathways (47). In H_2_O_2_-induced injury models, exendin-4-mediated upregulation of Nrf2, HO-1, and NQO1 was associated with reduced ROS levels and improved cell viability, while Nrf2 interference attenuated its protective effect; HO-1 overexpression likewise reduced oxidative and apoptotic injury in ARPE-19 cells (48, 49). In HG-ARPE-19 models, fat mass and obesity-associated protein (FTO)-mediated N6-methyladenosine (m6A) regulation and general control nonderepressible 2 (GCN2) knockdown further implicate Nrf2/HO-1-related signaling in the control of RPE oxidative stress and apoptosis (50, 51).

### 3.4 Retinal endothelial cells and the neurovascular unit

Retinal microvascular endothelial cells maintain the selective permeability and integrity of the inner blood–retinal barrier (BRB) through specialized tight junctions, thereby supporting the retinal microenvironment required for neuronal function. In OHT models, endothelial mitochondrial fragmentation, increased oxidative stress, and inner BRB leakage can precede or accompany early RGC degeneration (52). In cultured human retinal endothelial cells (HRECs), dimethyl fumarate (DMF) activates Nrf2/HO-1, reduces ROS production, and improves cell survival under HG conditions, suggesting that this axis may participate in adaptive endothelial protection against HG-induced oxidative and metabolic stress (53). In diabetic or HG-related endothelial injury models, 20(R)-ginsenoside Rg3 (R-Rg3) attenuated endothelial barrier disruption and stress-related injury, with ML385 intervention in HRECs supporting Nrf2 involvement in its *in vitro* endothelial protective effect (54). In primary human retinal microvascular endothelial cells (HRMECs) and retinal angiogenesis models, RS9-related Nrf2 activation is associated with reduced endothelial migration, hyperpermeability, and pathological vascular instability (55). By contrast, Ganoderma lucidum polysaccharides (GLP)-mediated protection in retinal ischemia–reperfusion (RIR) models appears more consistent with NVU-level than purely endothelial-specific regulation, with Nrf2/HO-1 upregulation paralleling reduced glial reactivity, vascular/NVU abnormalities, and functional impairment (56).

### 3.5 Müller glia and microglia

Müller cells and microglia represent two major glial components linking retinal homeostasis to oxidative–inflammatory responses. Müller cell-focused evidence shows that Müller cell-specific Nrf2 deficiency in oxygen-induced retinopathy (OIR) exacerbates pathological neovascularization, gliosis, and dysregulation of inflammatory/angiogenic transcriptional programs (57). In sodium iodate (NaIO_3_)-induced injury in the human Müller cell line MIO-M1, selected Lycii Fructus–Chrysanthemum Flos (LF–CF) extracts upregulated Nrf2/HO-1 and attenuated Müller cell injury, while ML385 weakened their cell-protective effect, supporting Nrf2 involvement in this glial context (58).

Evidence from microglial studies mainly centers on modulation of oxidative–inflammatory phenotypes. In RIR models, roflumilast attenuated Iba-1^+^ microglial activation and promoted a shift toward an M2-like phenotype, accompanied by increased Nrf2 and HO-1/NQO1 expression, reduced stimulator of interferon gene (STING)/NF-κB activation, and attenuation of these effects after Nrf2 inhibition or knockdown (59). In experimental OHT rats and lipopolysaccharide (LPS)-stimulated BV2 microglia/R28 retinal precursor cell co-culture systems, the sirtuin 6 (SIRT6) activator MDL800 modulated microglial inflammatory polarization, in association with Nrf2/HO-1-related signaling, reduced RGC apoptosis, and improved R28 cell survival (60).

The cell type-specific protective actions associated with Nrf2/HO-1 signaling in RNDs are summarized in Figure 2.

**Figure 2:**
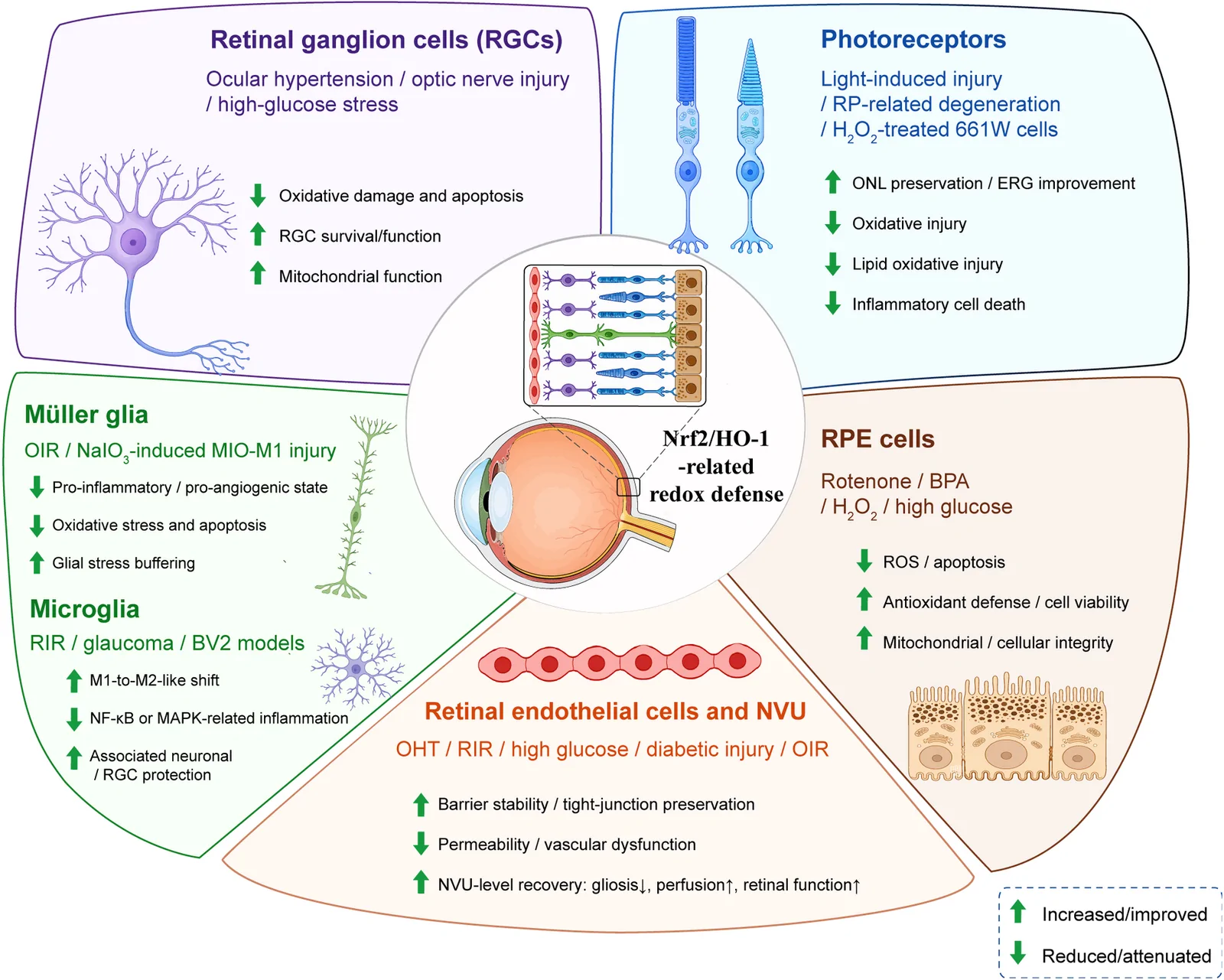
Cell type-specific protective phenotypes associated with Nrf2/HO-1 signaling in RNDs. Nrf2/HO-1-associated responses are summarized across major retinal cell types and NVU-related compartments, including RGCs, photoreceptors, RPE cells, retinal endothelial cells, Müller glia, and microglia. In preclinical RND-related contexts, these responses are generally associated with reduced oxidative damage, apoptosis, lipid oxidative injury, inflammatory signaling, vascular permeability, and glial stress responses, as well as improved RGC survival/function, ONL preservation, ERG responses, RPE cellular integrity, barrier stability, and NVU-level recovery. Upward and downward arrows indicate increased/improved and reduced/attenuated outcomes, respectively.

## 4 Redox-centered protective mechanisms associated with Nrf2/HO-1 signaling in RNDs

### 4.1 Redox homeostasis, mitochondrial integrity, and cell-death control

#### 4.1.1 Nrf2-driven antioxidant enzyme reprogramming

At low levels, ROS/RNS participate in cellular signaling; however, excessive production or insufficient clearance can disrupt redox homeostasis and damage cellular components, including proteins, lipids, and DNA (61). In RND-related retinal pathological contexts, this imbalance is often accompanied by impaired antioxidant defense, disruption of mitochondrial homeostasis, and dysregulated Nrf2/ARE adaptive responses (4). Owing to its high energy demand, chronic photic stimulation, and active mitochondrial metabolism, the retina is particularly vulnerable to redox imbalance (3, 4). Therefore, the Nrf2/HO-1 axis may serve as an important link between redox stress responses and regulation of the antioxidant enzyme system.

In RND-related cellular models of oxidative injury, Nrf2/HO-1-associated antioxidant defense reprogramming is mainly reflected by induction of ARE-related antioxidant molecules, reduction of oxidative burden, and restoration of cellular antioxidant capacity. In H_2_O_2_-induced oxidative injury in ARPE-19 cells, wheat alkylresorcinols enhanced the expression of antioxidant molecules, including CAT, GCLC/GCLM, NQO1, and HO-1, and reduced ROS generation; Nrf2 knockdown attenuated HO-1 induction and cytoprotection (62). Thymoquinone also reduced H_2_O_2_-induced ROS/MDA elevation, restored GSH and SOD levels, and enhanced Nrf2/HO-1 signaling; Nrf2 silencing weakened or reversed its protective effects against oxidative injury and apoptosis (63).

Beyond classical Nrf2 responses induced by oxidative stimuli, Nrf2/HO-1-related defense reprogramming may also be influenced by m6A epitranscriptomic regulation, chromatin-remodeling factors, and upstream signals such as ERK1/2. In HG-ARPE-19 models, FTO enhanced Nrf2/HO-1 signaling by reducing m6A modification of Nrf2 mRNA, thereby alleviating ROS/MDA elevation, glutathione redox imbalance, and apoptotic responses; knockdown of Nrf2 or HO-1 weakened its protective effects (50). Curcumin promoted Nrf2 nuclear translocation and increased HO-1 activity through an ERK1/2-related mechanism, while attenuating loss of cell viability and caspase-3-associated apoptotic responses (64). In RGC-related stress models, BRG1 overexpression or nobiletin treatment enhanced Nrf2/HO-1 signaling and reduced HG-, hypoxia-, or OHT-associated oxidative injury and apoptosis; Nrf2 silencing or HO-1 inhibition further supported pathway involvement in these protective effects (34, 40).

#### 4.1.2 Mitochondrial integrity, bioenergetics, and ROS containment

Mitochondria are central to ATP generation, mtROS production, redox compartmentalization, and mitochondrial ROS handling. Mitochondrial dysfunction can aggravate retinal cell injury through impaired oxidative phosphorylation, ATP insufficiency, and ROS amplification (65). Studies related to AMD and glaucoma suggest that mitochondrial injury varies across diseases and cell types, but commonly manifests as bioenergetic impairment, ROS amplification, and abnormalities in mitochondrial dynamics or quality-control processes such as mitophagy (66–68).

The protective role of Nrf2 in mitochondrial homeostasis is not limited to the direct induction of canonical antioxidant enzymes. In oxidatively stressed RPE cells, Nrf2 deficiency impairs the expression of pentose phosphate pathway-related genes and IDH2-associated NADPH supply, and reduces the reductive capacity of the TRX2/PRX3 system, thereby limiting mitochondrial H_2_O_2_ clearance and aggravating mitochondrial membrane potential loss and respiratory dysfunction (69). In blue light-exposed Sprague–Dawley rat retinas and ARPE-19 cell models, a xanthophyll-rich marigold extract formulation (BlingLife®-marigold extract; BLM) attenuated mitochondrial damage and oxidative stress; in ARPE-19 cells, ML385 weakened this protection, providing *in vitro* pharmacological support for Nrf2 involvement in BLM-associated cytoprotection (70). In an H_2_O_2_-induced apoptotic model of ARPE-19 cells, lentivirus-mediated HO-1 overexpression improved mitochondrial membrane potential, reduced ROS levels, and downregulated caspase-3 expression, accompanied by Nrf2 nuclear translocation and increased Bcl-2 expression (49). Collectively, these RPE-focused findings suggest that Nrf2/HO-1-related responses may help preserve mitochondrial membrane potential, limit ROS amplification, and reduce apoptosis under oxidative mitochondrial stress.

#### 4.1.3 Lipid peroxidation, iron handling, and ferroptotic vulnerability

In RPE-related degenerative injury, disruption of iron homeostasis and lipid peroxidation can aggravate oxidative stress and increase cellular susceptibility to ferroptosis. In a NaIO_3_-mediated RPE oxidative stress/AMD-related model, SLC12A3 inhibition or knockdown further exacerbated RPE injury, accompanied by increased Fe^2+^ and MDA, decreased GSH, reduced GPX4, and upregulated Nrf2/HO-1, suggesting that Nrf2/HO-1 elevation in this ferroptosis-prone context may reflect an insufficient compensatory stress response rather than effective protective activation (71). In HG-ARPE-19 models, tectochrysin reduced ROS/MDA and Fe^2+^ levels, restored GSH, reversed ACSL4/GPX4 abnormalities, and was accompanied by reactivation of Nrf2/HO-1 signaling (72). Together, these findings place Nrf2/HO-1-related responses at the interface of iron handling, lipid peroxidation, and GPX4-related ferroptotic vulnerability in RPE degenerative injury, while the SLC12A3 model cautions against interpreting pathway upregulation as effective cytoprotection without context-specific validation.

### 4.2 Redox–inflammatory crosstalk in RNDs

#### 4.2.1 Redox-sensitive inflammatory signaling and cytokine responses

In RND-related stress contexts, oxidative stress not only directly causes molecular damage but may also sustain inflammatory responses through the release of damage-associated molecular patterns (DAMPs), cellular senescence/senescence-associated secretory phenotype (SASP), and redox-sensitive inflammatory pathways such as NF-κB (26). The Keap1/Nrf2/HO-1 axis may therefore function as an adaptive protective system linking oxidative stress defense with inflammatory regulation (18, 26). Glial responses can amplify retinal redox–inflammatory crosstalk; related cell type-specific evidence has been discussed in Section 3. This section therefore focuses on inflammatory signaling and its injury-related consequences.

In RIR, experimental glaucoma, and DR-related retinal injury models, evidence linking Nrf2/HO-1-associated antioxidant responses with changes in inflammatory signaling is relatively concentrated. Resveratrol (RSV) increased RGC survival and improved ERG responses, while downregulating NLRP3, ASC, cleaved caspase-1, and IL-1β and modulating the Keap1/Nrf2/HO-1 pathway (37). In a rabbit RIR model induced by OHT, epigallocatechin 3-gallate (EGCG) attenuated early retinal oxidative injury, glial responses, and cell-death markers, accompanied by Nrf2 nuclear translocation and increased HO-1 expression (73). In STZ-related diabetic rat retinal models, rhaponticin partially restored Nrf2/NQO1/HO-1 expression, downregulated Keap1, and reduced MDA levels; these changes were accompanied by decreased NF-κB, TNF-α, and MMP-2, together with increased IL-10 and TIMP-1 (74).

Light injury and exogenous toxicant models further suggest that crosstalk between Nrf2/HO-1-related redox regulation and inflammatory responses can occur across different stress contexts. In UV-induced ARPE-19 injury, increased ROS, decreased SOD1/CAT, and elevated TNF-α, IL-1β, and IL-6 were partially alleviated by active components of Dendrobium nobile, accompanied by modulation of Nrf2/HO-1 and MAPK signaling (75). In LED-induced retinal injury, redox imbalance, inflammatory cytokine elevation, reduced retinal Nrf2/HO-1 expression, and upregulation of NF-κB, intercellular adhesion molecule-1, glial fibrillary acidic protein (GFAP), and monocyte chemoattractant protein-1 were partially reversed by combined lutein/zeaxanthin (L/Z) and quercetagetin (QCG) treatment (76). 2-ethylhexyl diphenyl phosphate induced intracellular and mitochondrial ROS elevation in 661W photoreceptor-like cells, accompanied by upregulation of Nrf2/HO-1/CAT and TNF-α, IL-1β, and IL-6, providing *in vitro* evidence that Nrf2/HO-1 responses and pro-inflammatory activation can occur in parallel under exogenous oxidative stress (77).

Overall, these studies suggest that Nrf2/HO-1-related responses are repeatedly coupled with ROS burden, NF-κB/NLRP3 signaling, and cytokine responses, supporting a role for this axis in redox–inflammatory crosstalk across RND-related injury contexts.

#### 4.2.2 Inflammation-associated neuronal injury in RNDs

In RND-related models, redox imbalance and inflammatory activation often coexist with retinal neuronal injury, including photoreceptor or RGC loss, and contribute to structural degeneration. In light-induced retinal degeneration, intense light exposure is associated with oxidative stress, photoreceptor death, NF-κB/NLRP3 inflammasome signaling, and pyroptosis-related pathways. Under injury conditions, HMGB2 translocates from the nucleus to the cytoplasm and is released extracellularly, accompanied by reduced Nrf2/HO-1 expression and increased IL-1β. HMGB2 knockdown enhances Nrf2/HO-1 expression, reduces pyroptosis-related proteins, and attenuates photoreceptor loss and ONL thinning, suggesting that HMGB2-associated suppression of Nrf2/HO-1 antioxidant defense is linked to NF-κB/NLRP3-related inflammatory photoreceptor death (43).

Related evidence has also been reported in a thyroxine/tail-clamping–preconditioned, NaIO_3_-induced dry AMD-like retinal injury model, in which oxidative stress, inflammatory cytokine elevation, retinal structural disruption, and apoptosis-related signaling occurred together. JuJing formula treatment increased Nrf2 and HO-1 expression, reduced IL-1β, IL-6, TNF-α, and cleaved caspase-3, modulated Bax/Bcl-2-related apoptotic signaling, and was associated with improved retinal structure and reduced apoptosis-related injury. These findings support an association among Nrf2/HO-1-related antioxidant responses, inflammatory cytokine attenuation, and reduced structural retinal injury in a dry AMD-like context (78).

In optic nerve crush models, increased ROS and inflammatory gene expression are accompanied by RGC loss and visual dysfunction. Post-injury administration of the Sig1R agonist (+)-Pentazocine (PTZ) upregulates Nrf2, HO-1, and NQO1, reduces IL-1β, TNF-α, and iNOS expression, and is associated with increased RGC survival, attenuation of GFAP-labeled glial responses, and improved visual function. In Sig1R-deficient mice, however, the structural and functional protective effects of (+)-PTZ are no longer evident, suggesting that these effects depend on Sig1R and are associated with antioxidant gene upregulation and inflammatory gene downregulation (79).

### 4.3 Nrf2/HO-1 in retinal vascular protection and NVU homeostasis

#### 4.3.1 Endothelial redox defense and BRB barrier maintenance

RIR injury is commonly used to model ischemic components relevant to glaucoma, DR, and other ischemia-related retinal disorders, and is often associated with oxidative stress, inflammation, neuronal injury, and altered vascular permeability (80, 81). In diabetic IOP-RIR models, diabetes exacerbates post-ischemic retinal injury and delays BRB recovery. Mouse IOP-RIR studies link increased vascular permeability to loss or mislocalization of inner BRB tight-junction proteins at endothelial borders, whereas rabbit RIR studies show that BRB leakage increases with ischemia duration (82, 83). Together, these findings identify vascular leakage and endothelial junctional disruption as key barrier-related consequences of ischemic retinal injury, particularly under diabetic stress.

Under diabetic and HG conditions, available evidence links Nrf2/HO-1-related endothelial protection to redox restoration, barrier preservation, and reduced cell death. In high-fat diet/streptozotocin (HFD/STZ)-induced type 2 diabetes mellitus (T2DM) mice and HG-treated HRECs, R-Rg3 enhanced Nrf2/HO-1 signaling, reversed the reduction in barrier proteins such as ZO-1 and occludin, and attenuated ROS-associated endoplasmic reticulum (ER) stress and apoptosis; in HRECs, ML385 weakened the endothelial protective effect of R-Rg3, providing pharmacological support for Nrf2 involvement in this *in vitro* model (54). In db/db mice and HG-treated HRMECs, gypenoside A-loaded nanoparticles (GPA-NPs) reduced oxidative injury, iron accumulation, and ferroptotic endothelial injury, accompanied by Keap1–Nrf2/HO-1/GPX4-related changes (13). In HG-treated HRECs, metoprolol also reduced ROS accumulation, promoted Keap1/Nrf2/HO-1-related responses, and was accompanied by inhibition of the ERK1/2–cPLA2–COX-2 axis and downregulation of TNF-α, IL-1β, and vascular endothelial growth factor (VEGF) mRNA (84). Overall, these studies link Nrf2/HO-1-related responses to endothelial redox defense in diabetes-related retinal microvascular injury, with potential implications for BRB maintenance.

#### 4.3.2 Pathological angiogenesis and NVU-level remodeling

Pathological angiogenesis and vascular remodeling in RNDs involve coordinated changes within the NVU, including neurons, glial cells, pericytes, and endothelial cells. Anatomical studies have shown that retinal vascular plexuses exhibit layer-specific patterns of glial ensheathment and neurovascular contacts. In the rd10 retinal degeneration model, Müller glial ensheathment around the deep vascular plexus is disrupted, suggesting that photoreceptor degeneration may be accompanied by compartment-specific NVU structural changes (85). This anatomical context helps frame pathological angiogenesis, vascular leakage, and post-ischemic remodeling as NVU-level processes rather than isolated endothelial events.

Within this NVU context, Nrf2/HO-1-related redox regulation may contribute to limiting pathological vascular remodeling and maintaining vascular integrity. In OIR models, nattokinase reduced neovascularization and FITC-dextran vascular leakage, accompanied by Nrf2/HO-1 upregulation and downregulation of pro-angiogenic and inflammatory factors. Brusatol weakened its anti-neovascular effect, providing *in vivo* pharmacological support for Nrf2/HO-1 involvement, while Nrf2 siRNA provided additional *in vitro* genetic loss-of-function support for Nrf2 involvement in NK-mediated suppression of human umbilical vein endothelial cells (HUVECs) angiogenic behaviors (86). The Nrf2 activator RS9 also inhibited pathological neovascularization in OIR models, reduced VEGF expression, and upregulated HO-1, PDGFR-β, and junction-associated molecules, including VE-cadherin, ZO-1, and claudin-5 (55). Quercetin-loaded tetrahedral framework nucleic acids (tFNAs-QUE) also reduced pathological neovascularization and retinal nonperfusion areas while preserving retinal neuronal structure and electrophysiological function, accompanied by upregulation of AKT/Nrf2/HO-1-related signaling (87). In RIR rats, GLP treatment was associated with Nrf2/HO-1 upregulation, attenuation of oxidative stress and glial responses, improved OCTA perfusion, downregulation of VEGF/HIF-1α/Notch signaling, and modulation of VE-cadherin/eNOS and Cx43/AQP4-related NVU markers, suggesting a possible role in post-ischemic vascular remodeling and NVU-level regulation (56).

The redox-centered inflammatory and neurovascular mechanisms associated with Nrf2/HO-1 signaling are summarized in Figure 3.

**Figure 3:**
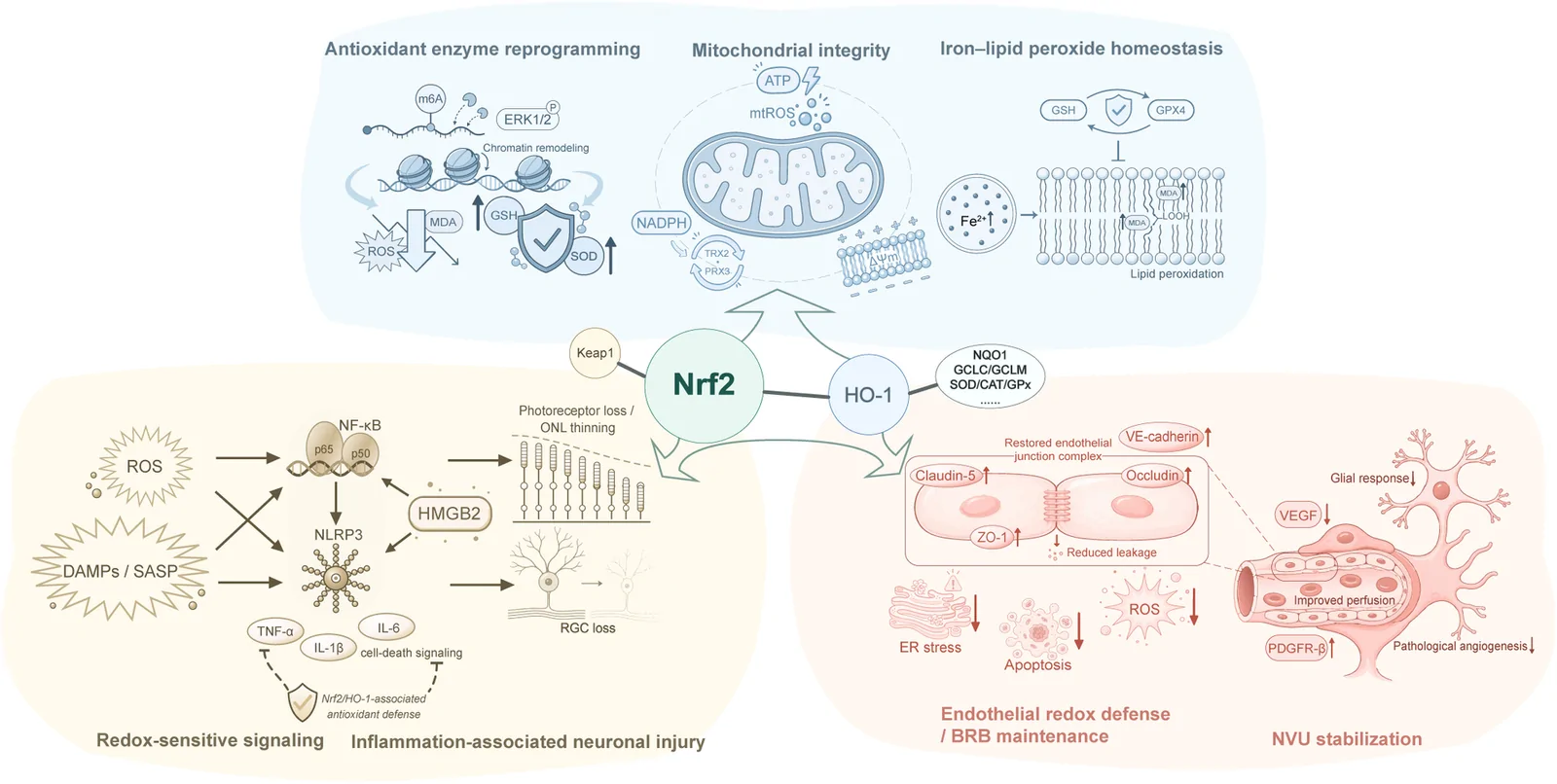
Nrf2/HO-1-associated redox-inflammatory regulation and neurovascular homeostasis in RNDs. Nrf2/HO-1-related responses are positioned at the interface of antioxidant enzyme reprogramming, mitochondrial integrity, iron–lipid peroxide homeostasis, redox-sensitive inflammatory signaling, and NVU regulation under retinal stress conditions. Through interactions with NF-κB/NLRP3-, HMGB2-, and cytokine-related pathways, this axis may modulate inflammatory processes associated with photoreceptor loss, ONL thinning, RGC injury, and other inflammation-associated neuronal changes. In endothelial and NVU-related compartments, Nrf2/HO-1-associated responses are linked to BRB maintenance, tight-junction preservation, reduced leakage and vascular dysfunction, improved perfusion, and modulation of pathological angiogenesis. Arrows and blunt-ended lines indicate activating/promoting and inhibitory/attenuating relationships, respectively.

### 4.4 Context-dependent interpretation of Nrf2/HO-1 signaling

Although the evidence reviewed above generally supports adaptive or protective roles for Nrf2/HO-1 signaling in RND-related models, pathway activation should not be equated with effective cytoprotection. The functional meaning of Nrf2/HO-1 upregulation varies with retinal cell type, disease stage, injury paradigm, activation kinetics, and evidence depth. Thus, Nrf2/HO-1 upregulation may indicate protective pathway involvement when supported by genetic or pharmacological validation, but it may also reflect insufficient compensatory stress signaling in models with ongoing injury.

HO-1-linked iron handling illustrates this context dependence. Under adaptive conditions, HO-1-mediated heme catabolism is coupled to iron-buffering mechanisms such as ferritin and contributes to redox protection (16). In ferroptosis-prone RPE injury, however, HO-1-related responses require cautious interpretation. In a MERTK-deficient model using donor-derived primary human RPE, silencing NFE2L2 or HMOX1 reduced Fe^2+^ accumulation and restored RPE phagocytosis and cytoskeletal organization (88). In the NaIO_3_/SLC12A3-related AMD-like model, Nrf2/HO-1 upregulation occurred alongside aggravated RPE and outer retinal injury and ferroptosis-related abnormalities, suggesting insufficient compensation (71). Conversely, other RPE and retinal vascular models link Nrf2/HO-1 activation and GPX4 preservation with reduced ferroptotic injury, indicating that the net effect of this axis depends on the accompanying iron-handling and lipid-peroxidation context (13, 72).

Similar caution applies to angiogenic, NVU-related, and inflammatory retinal microenvironments. As summarized in Sections 4.2 and 4.3, Nrf2/HO-1 activation is often associated with reduced inflammatory signaling, glial activation, vascular leakage, pathological neovascularization, or NVU disruption (37, 55, 56, 84, 86, 87). These associations should not be generalized across disease stages or retinal compartments. Current retinal evidence does not establish that sustained Nrf2/HO-1 activation directly promotes pathological angiogenesis, but angiogenic settings require separate evaluation of activation kinetics, endothelial responses, and NVU-level outcomes. Moreover, in EHDPP-exposed 661W cells, Nrf2/HO-1/CAT upregulation coexisted with increased TNF-α, IL-1β, and IL-6, indicating that pathway activation does not necessarily imply inflammatory control (77).

Collectively, Nrf2/HO-1 signaling in RNDs should be interpreted as a context-dependent stress-response axis. Recognizing these distinctions may help avoid overgeneralizing Nrf2/HO-1 activation as uniformly protective and support a more cautious assessment of its therapeutic relevance across retinal disease models.

## 5 Disease-specific evidence in RNDs

### 5.1 Diabetic retinopathy

In DR, Nrf2/HO-1-related evidence is mainly derived from preclinical models of vascular barrier injury, microvascular endothelial ferroptosis, and RGC damage. In HFD/STZ-induced mice and HG-treated HRECs, R-Rg3 provided DR-related endothelial barrier-protection evidence, with *in vitro* ML385 data supporting Nrf2 involvement in its endothelial protective effects (54). In diabetic rats, rhaponticin was associated with upregulation of Nrf2/NQO1/HO-1 and attenuation of oxidative stress, inflammation, and histological abnormalities (74). GPA-NPs further extend DR-related evidence to microvascular endothelial ferroptosis (13). At the RGC level, RSV improved retinal morphology and pattern ERG responses and reduced RGC apoptosis in STZ-induced diabetic mice; its protective effect in HG-treated RGCs was weakened by Nrf2 interference, supporting the involvement of Nrf2-related signaling in preclinical protection against diabetic neuronal injury (35).

### 5.2 Glaucoma and optic neuropathy

In glaucoma-related models, available Nrf2/HO-1-related evidence remains preclinical and spans chronic OHT and acute OHT/RIR paradigms, primarily concerning RGC antioxidant defense, apoptosis regulation, structural preservation, and cell survival. In rats with chronic OHT, elevated IOP was accompanied by RGC loss and stress-related upregulation of Nrf2/HO-1 expression; tBHQ further increased Nrf2/HO-1 expression and reduced RGC apoptosis, suggesting an association between enhanced Nrf2/HO-1 signaling and attenuation of chronic OHT-related RGC injury (36). AUF1 overexpression reduced RGC apoptosis in an acute OHT/retinal ischemic injury model and was accompanied by increased Nrf2/HO-1 expression, suggesting that this axis is associated with regulation of RGC apoptosis under acute injury conditions (89). In an acute OHT-induced RIR model, astaxanthin improved retinal structure and RGC survival and attenuated the Bax/Bcl-2 imbalance; these effects were weakened under Nrf2-deficient conditions, consistent with the involvement of Nrf2-related anti-apoptotic protection of RGCs (39). In OHT and hypoxic RGC models, nobiletin attenuated RGC apoptosis, oxidative stress, and inner retinal injury, and its protective effects were weakened by the HO-1 inhibitor SnPP, further supporting the involvement of HO-1-related responses in RGC protection (40). MDL800-related evidence further suggests a link between glaucoma-related RGC protection and modulation of the microglial inflammatory milieu (60).

In optic nerve crush (ONC) and traumatic optic neuropathy (TON)-related optic nerve injury models, Nrf2/HO-1-related signaling is also associated with RGC survival and preservation of visual function after axonal injury. Dimethyl fumarate increased the density of tubulin β3-positive RGCs after ONC and improved the positive scotopic threshold response, accompanied by enhanced Nrf2/HO-1 signaling in RGCs (33). The Sig1R agonist (+)-PTZ improved RGC-related structural and functional outcomes after ONC, including pattern ERG (PERG), visual evoked potential (VEP), and retinal/vascular imaging indices, accompanied by Nrf2/HO-1/NQO1 upregulation (79). Regarding endogenous regulation, PARK7 downregulation aggravated RGC loss, photopic negative response (PhNR) decline, and optomotor response impairment after ONC, accompanied by increased Keap1 and decreased Nrf2/HO-1 (41). In a rat ONC model used to simulate TON, intravitreal PEGylated granulocyte colony-stimulating factor (PEG-G-CSF) preserved flash VEP (FVEP) responses and RGC survival and reduced apoptosis and inflammatory cell infiltration, accompanied by Nrf2/HO-1-related changes in optic nerve tissues (90).

### 5.3 AMD and RPE degeneration

In AMD-related studies, available Nrf2/HO-1 evidence is concentrated in non-neovascular or dry AMD-like models of RPE and outer retinal degeneration, mainly involving oxidative/carbonyl stress, mitochondrial injury, ferroptosis, and apoptosis. In NaIO_3_ models, glycyrrhizin attenuated RPE/retinal ROS accumulation, apoptosis, retinal thinning, and functional impairment, accompanied by Akt–Nrf2/HO-1-related signaling enhancement (91). Morin hydrate reduced lipid accumulation, ER stress, and oxidative stress in CSE-induced AMD-like injury in RPE cells and the mouse RPE layer, accompanied by AMPK–Nrf2–HO-1-related signaling (92). In A2E/blue light-exposed ARPE-19 models, *Codonopsis lanceolata* attenuated 4-HNE-related carbonyl stress and cell death, accompanied by regulation of Keap1/Nrf2/HO-1 and Bcl-2 family proteins (93). In addition, extracts of the LF–CF herbal pair reduced oxidative injury and apoptosis in NaIO_3_-induced AMD-like retinal degeneration and MIO-M1 cells, suggesting that Nrf2/HO-1-related antioxidant responses may also be relevant in Müller glial contexts (58). In NaIO_3_-challenged ARPE-19 cells and mouse RPE, SLC12A3 inhibition or knockdown aggravated redox imbalance, mitochondrial injury, and ferroptotic features despite increased Nrf2/HO-1 expression and reduced GPX4, highlighting the context-dependent interpretation of pathway upregulation in ferroptosis-prone RPE injury (71).

Human-relevant evidence remains limited. Patient-derived RCBTB1-deficient iPSC-RPE showed mitochondrial dysfunction and attenuated oxidative stress-induced NFE2L2 expression, whereas metformin or TN1 reduced apoptosis in NaIO3-injured human retinal organoids, accompanied by increased HMOX1 expression. However, neither the RCBTB1-deficient phenotype nor the drug-associated protection was shown to be causally dependent on NFE2L2 or HMOX1, respectively, in these human-derived systems (94, 95).

### 5.4 Retinal ischemia–reperfusion and hypoxia-driven injury models

In classic RIR injury models, Nrf2/HO-1-related evidence mainly points to RGC or retinal neuronal protection, oxidative-stress suppression, and attenuation of cell death. In an IOP elevation-induced mouse RIR model, RSV improved Brn3a-positive RGC survival and ERG responses, with pharmacological intervention supporting the involvement of Keap1/Nrf2/HO-1 and inflammasome-related signaling in this protective context (37). EGCG attenuated early tissue injury, GFAP-positive glial responses, lipid peroxidation, and TUNEL/HMGB1-related injury signals in a rabbit RIR model, while enhancing Nrf2 nuclear translocation and HO-1 expression (73). Brazilian green propolis increased Brn3a-positive RGC survival in a mouse RIR model, accompanied by upregulation of Nrf2/HO-1 and downregulation of apoptosis-related signals such as Bax/p53 and NF-κB-related inflammatory signaling (96). In addition, N-acetylserotonin (NAS) combined with aflibercept provided combination-therapy evidence in RIR-injured rats, with retinal protection associated with reduced apoptosis and oxidative stress and enhanced Akt/Nrf2/HO-1 signaling (97). GLP also provided RIR-specific evidence of NVU-level protection, with reduced oxidative stress, apoptosis, gliosis, and vascular dysfunction accompanied by increased Nrf2/HO-1 expression (56).

In hypobaric hypoxia and OIR/retinopathy of prematurity (ROP) models, Nrf2/HO-1-related evidence expands from neuronal injury protection to pathological neovascularization, vascular leakage, and NVU-level regulation. Hesperidin restored the hypobaric hypoxia-induced reduction of Nrf2/HO-1 expression and attenuated TUNEL-positive cells and mitochondria-related apoptotic changes involving caspase-3/9 and Bax/Bcl-2 (98). In OIR models, nattokinase reduced pathological neovascularization and vascular leakage; brusatol weakened its anti-neovascular effect, providing *in vivo* pharmacological support for Nrf2/HO-1 involvement, with additional *in vitro* Nrf2-siRNA support in endothelial angiogenic assays (86). In ROP/OIR models, melatonin-related protection was associated with miR-23a-3p/Nrf2 modulation, upregulation of Nrf2, HO-1, and antioxidant enzymes, and reductions in VEGF, MDA, and apoptosis (99).

### 5.5 Photoreceptor degeneration: retinitis pigmentosa and light-induced retinal injury

In models of RP and light-induced retinal degeneration, Nrf2/HO-1-related evidence mainly points to preservation of outer retinal structure, attenuation of photo-oxidative and inflammatory stress, and, in some models, maintenance of retinal function. In rd10 mice, Fructus Lycii–Salvia miltiorrhiza Bunge extracts increased ONL thickness and cell number, improved scotopic ERG a- and b-waves, and were accompanied by increased SOD and GSH-Px, reduced MDA, and upregulation of Nrf2/HO-1, suggesting that this axis may participate in antioxidant protection in RP (44). Notably, in a donor-derived primary human RPE model of MERTK deficiency, silencing either NFE2L2 or HMOX1 reduced Fe^2+^ accumulation and restored cytoskeletal organization and RPE phagocytosis of photoreceptor outer segments, supporting functional involvement of both molecules in RPE dysfunction in this genetic context (88). In light-injury models, HMGB2 translocation/release was associated with Nrf2/HO-1 downregulation, redox-inflammatory pathway activation, and photoreceptor death; HMGB2 knockdown preserved the ONL and increased Nrf2/HO-1 expression, supporting a link between impaired antioxidant defense and inflammatory photoreceptor injury (43). A blue light injury study further showed that BLM improved retinal histopathological changes and attenuated mitochondrial oxidative stress and senescence-related changes in rat retinal tissue and ARPE-19 cells; ML385 weakened its protective effect in ARPE-19 cells, providing *in vitro* pharmacological support for Nrf2 involvement in RPE cytoprotection (70). In a separate LED-induced photo-oxidative retinal injury model, L/Z combined with QCG preserved ONL thickness and restored GAP43 and NCAM expression, alongside Nrf2/HO-1-related redox modulation, supporting an association with preservation of outer retinal structure and neuroplasticity markers (76).

Because the studies summarized in this review differ substantially in model system, intervention/manipulation, and depth of pathway validation, Nrf2/HO-1-related evidence levels were assigned according to the directness of pathway-specific support. Level 1 denotes direct genetic manipulation of Nrf2 or HO-1 with phenotype-level validation. Loss-of-function or knockout experiments that attenuated a protective phenotype were interpreted as supporting pathway necessity, whereas overexpression provided genetic gain-of-function evidence for functional involvement; rescue experiments were considered to provide stronger causal support. Level 2 denotes pharmacological support for pathway involvement when pathway inhibition attenuated the reported protective phenotype, with inhibitor specificity considered where uncertain. Level 3 indicates pathway activation associated with protection without reverse validation; Level 4, expression or pathological association only; Level 5, upstream regulator-associated evidence without direct Nrf2/HO-1 validation; and Level 6, context-dependent, delivery-related, combination-related, or non-protective associations. These levels indicate pathway-specific support rather than overall therapeutic efficacy or translational readiness.

Using this framework, a structured summary of representative disease- and model-specific evidence and corresponding Nrf2/HO-1-related evidence levels is provided in Table 1.

**Table 1:** Representative disease-specific evidence associated with Nrf2/HO-1 signaling in RNDs.

Ref.	Model/system	Main retinal target	Intervention/manipulation	Nrf2/HO-1 evidence level	Key molecular/pathological changes	Main structural/functional outcomes
Diabetic retinopathy/diabetic retinal neurovascular injury						
(54)	HFD/STZ-induced T2DM mouse model + HG-treated HRECs	Retinal endothelial cells/iBRB	R-Rg3; ML385 for *in vitro* Nrf2-inhibition validation	Level 2: *in vitro* pharmacological support for Nrf2 involvement; ML385 attenuated R-Rg3-associated endothelial protection.	Reduced oxidative/ER stress, apoptosis, and endothelial barrier disruption.	Retinal permeability, thickness changes, and histopathological injury were alleviated; HG-induced HREC barrier injury was reduced.
(74)	Nicotinamide/STZ-induced diabetic rat model	Whole retina; no cell-specific validation	Rhaponticin	Level 3: Pathway activation associated with protection; no inhibitor/genetic validation.	Enhanced antioxidant defense; reduced lipid peroxidation and NF-κB-related inflammation.	Retinal edema-like changes, layer disorganization, and IPL neovascular changes were ameliorated.
(13)	db/db type 2 diabetic mouse model + HG-treated HRMECs	Retinal microvascular endothelium/HRMECs	GPA-NPs; GPA-NPs in HG-treated HRMECs; erastin challenge	Level 5: Direct Keap1-targeting/upstream evidence; Nrf2/HO-1/GPX4 activation occurred without Nrf2/HO-1 loss-of-function validation.	Reduced ferroptotic lipid injury, mitochondrial dysfunction, inflammation, and VEGF-related vascular stress.	Retinal fluorescein leakage, vascular disorganization, retinal pathology, and GCL/INL injury were reduced; HG-induced HRMEC ferroptotic injury was attenuated.
(35)	STZ-induced diabetic mouse model + HG-treated primary mouse RGCs	RGCs/inner retina	RSV; Nrf2 siRNA for *in vitro* validation	Level 1: *in vitro* Nrf2 loss-of-function evidence; Nrf2 siRNA attenuated RSV-associated cytoprotection in HG-treated primary RGCs.	Restored antioxidant defense and reduced oxidative stress and RGC apoptosis.	Retinal morphology, RGC density, and PERG P50/N95 responses were partly preserved.
Glaucoma and optic neuropathy/RGC degeneration						
(36)	Ocular venous ligation-induced rat COH glaucoma model	RGCs/inner retina	tBHQ after IOP elevation	Level 3: Pharmacological Nrf2 activation associated with protection; no reverse genetic/pharmacological validation.	Reduced RGC apoptosis.	GCL organization, IPL thickness, and FG-labeled RGC survival improved.
(39)	IOP elevation-induced WT and Nrf2−/− mouse RIR model	RGCs/inner retina	AST; Nrf2−/− mice for genetic validation	Level 1: *in vivo* Nrf2 knockout evidence; Nrf2 deficiency attenuated AST-mediated retinal/RGC protection.	Reduced oxidative stress and RGC apoptosis; improved antioxidant activity.	Retinal integrity/thickness and FG-labeled RGC survival were preserved.
(89)	Acute IOP elevation-induced mouse retinal ischemia model	RGCs/inner retina	AUF1 overexpression; MG132 co-treatment	Level 5: Upstream AUF1-associated evidence; MG132 co-treatment did not provide Nrf2/HO-1-specific validation.	Reduced apoptosis and improved cell survival.	Retinal thickness/layer integrity and RGC survival increased; RGC apoptosis decreased.
(33)	Mouse ONC model	RGCs/inner retina	DMF; Nrf2/HO-1 assessed in RGCs after ONC	Level 3: Nrf2/HO-1 activation associated with RGC protection; no loss-of-function validation.	Nrf2 nuclear expression and HO-1 upregulation in RGCs.	RGC survival and pSTR amplitude improved after ONC.
(79)	WT and Sig1R−/− mouse ONC model	RGCs/inner retina; Müller gliosis and retinal vasculature assessed	(+)-PTZ after ONC; WT and Sig1R−/− mice for Sig1R-dependence testing	Level 5: Upstream regulator-associated evidence; protection required Sig1R, without direct Nrf2/HO-1 validation.	Reduced oxidative stress, inflammation, and gliosis.	Visual function, retinal structure, vascular morphology, and RGC survival were partly preserved.
(60)	LPS-induced BV2 microglial inflammation model + BV2-R28 co-culture + anterior-chamber perfusion-induced rat OHT model	Retinal microglia; R28 cells in co-culture; RGCs/GCL *in vivo*	MDL800; SIRT6 inhibition/knockdown for validation	Level 5: Upstream SIRT6 loss-of-function support; SIRT6 inhibition/knockdown attenuated MDL800 responses without direct Nrf2/HO-1 validation.	Suppressed microglial M1-like inflammation, ROS and MAPK-related signaling, and R28 mitochondrial apoptosis.	R28 viability increased and apoptosis decreased; OHT-associated GCL/RGC loss, IPL thinning, and GCL apoptosis were partly reduced.
(40)	Microbead-induced rat OHT model + hypoxia-exposed primary RGCs	RGCs/inner retina; Müller glia assessed	NOB; SnPP for HO-1 inhibition validation	Level 2: *in vivo* pharmacological support for HO-1 involvement; SnPP attenuated NOB-associated RGC protection.	Reduced oxidative stress, Müller gliosis, and RGC apoptosis; no IOP-lowering effect.	RGC survival, inner retinal structure, and PhNR amplitude were partly preserved; hypoxia-induced primary RGC apoptosis decreased.
(41)	Mouse ONC model	RGCs/inner retina	Intravitreal rAAV-shRNA(PARK7)-EGFP before ONC	Level 5: Upstream PARK7 loss-of-function evidence; PARK7 silencing reduced Nrf2/HO-1 signaling without direct pathway manipulation.	PARK7 knockdown increased apoptosis/cell-death signaling.	PARK7 knockdown aggravated ONC-induced RGC loss and reduced PhNR amplitude and OMR-based visual acuity.
(90)	Rat ONC/traumatic optic neuropathy-like model	RGCs and optic nerve lesion site/RGC axons	Intravitreal PEG-G-CSF after ONC	Level 6: Long-acting delivery-related optic-nerve Nrf2/HO-1 association; no pathway-specific reverse validation.	Reduced RGC-layer apoptosis and ED1-positive lesion-site cells; increased intrinsic microglial activation.	RGC survival and FVEP P1-N2 amplitude were partly preserved after ONC.
AMD and RPE degeneration						
(71)	NaIO3-induced AMD-like mouse model + ARPE-19 oxidative-stress model with SLC12A3 knockdown/NCC inhibition	RPE cells; secondary photoreceptor/outer retinal injury	Clopamide with NaIO3 injury; AAV9-mediated RPE SLC12A3 knockdown; ARPE-19 SLC12A3 knockdown/inhibition	Level 6: Context-dependent/non-protective association; Nrf2/HO-1 upregulation accompanied worsened ferroptotic RPE/retinal injury without direct pathway reversal.	Increased oxidative stress, ferroptotic lipid/iron injury, mitochondrial dysfunction, and RPE cell death/migration impairment.	Clopamide worsened NaIO3-induced RPE damage, ZO-1 disruption, and ERG b-wave reduction; SLC12A3 knockdown aggravated retinal thinning/disorganization and photoreceptor marker loss.
(91)	NaIO3-induced atrophic AMD-like mouse model + NaIO3-treated ARPE-19 cells	RPE cells; outer retina/photoreceptor-associated injury	Glycyrrhizin pretreatment; LY294002 for upstream PI3K/Akt testing	Level 5: Akt–Nrf2/HO-1 upstream regulator-associated evidence; no direct Nrf2/HO-1 loss-of-function validation.	Reduced ROS and caspase-3-associated apoptosis in RPE/retinal injury.	Retinal thinning, ONL/INL disorganization, and drusen-like lesions were reduced; ERG a-/b-wave amplitudes were partly preserved.
(58)	NaIO3-induced dry AMD-like mouse model + NaIO3-treated MIO-M1 Müller cells	Müller cells/MIO-M1; GCL, INL, and ONL	LF–CF extracts; ML385 for *in vitro* Nrf2-inhibition validation	Level 2: *in vitro* pharmacological support for Nrf2 involvement; ML385 attenuated LF–CF-associated protection in NaIO3-treated MIO-M1 cells.	Reduced oxidative stress and caspase-dependent Müller-cell/retinal apoptosis; preserved antioxidant/glutamine-synthetase phenotype.	Retinal disorganization, cell loss, and GCL/INL/ONL thinning were attenuated.
(93)	A2E/blue-light-induced ARPE-19 model	RPE cells/ARPE-19 cells	Codonopsis lanceolata extract before A2E/blue-light injury; lutein comparator	Level 3: Pathway activation associated with protection; no inhibitor/genetic validation.	Reduced carbonyl stress and intrinsic apoptosis in A2E/blue-light-injured ARPE-19 cells.	ARPE-19 viability improved and apoptosis decreased.
(92)	CSE-treated human RPE cells + CSE intravitreal injection-induced AMD-like mouse model	RPE cells/RPE layer	MH in CSE models; Compound C for AMPK-inhibition validation	Level 5: Upstream AMPK-associated pharmacological evidence; Compound C attenuated MH-associated protection and Nrf2 activation without direct Nrf2/HO-1 validation.	Reduced oxidative/mitochondrial ROS stress, ER stress, and lipid/cholesterol accumulation.	RPE cytotoxicity/proliferation loss, RPE/BrM lipid-cholesterol deposition, and AMD-like histopathology were attenuated.
(94)	Patient-derived RCBTB1-deficient iPSC-RPE + healthy control iPSC-RPE; tBHP challenge	RPE cells/mitochondria	RCBTB1-deficient patient iPSC-RPE; tBHP oxidative-stress challenge	Level 5: Patient-derived RCBTB1-associated evidence; attenuated NFE2L2 stress response without direct pathway manipulation.	Increased ROS and oxidative-stress sensitivity; mitochondrial dysfunction and attenuated NFE2L2 induction.	Mitochondrial ultrastructure and membrane potential were impaired; POS phagocytosis was unchanged.
(95)	NaIO3-injured hESC-derived human retinal organoids + NaIO3-induced mouse retinal degeneration model	RPE and photoreceptors/outer retina	Metformin or TN1; concurrent treatment or 7-day pretreatment	Level 3: Metformin/TN1-associated HMOX1 upregulation with protection; no NFE2L2/HMOX1 loss-of-function validation.	Reduced apoptosis and oxidative stress; increased HMOX1/antioxidant-gene expression and restored RPE/photoreceptor-related genes.	Organoid apoptosis decreased; mouse ONL structure and ERG a-/b-wave amplitudes were preserved.
Retinal ischemia/hypoxia-related injury						
(37)	IOP elevation-induced mouse retinal I/R model	RGCs/retinal tissue	RSV; ML385 for Nrf2 inhibition; Nigericin/MCC950 for NLRP3 modulation	Level 2: *in vivo* pharmacological support for Nrf2 involvement; ML385 attenuated Res-associated RGC and ERG protection.	Suppressed NLRP3 inflammasome/IL-1β inflammation and activated Keap1/Nrf2/HO-1 signaling.	RGC survival and ERG a-/b-wave amplitudes improved after retinal I/R.
(73)	IOP elevation-induced rabbit retinal I/R model	Retinal neuronal layers, mainly GCL and INL	EGCG	Level 3: Nrf2 nuclear translocation/HO-1 upregulation associated with early retinal protection; no reverse validation.	Reduced lipid peroxidation, gliosis, apoptosis, and HMGB1-positive necrosis.	Retinal histological damage and early neuronal cell death were reduced; GCL/IPL/INL organization was better preserved.
(86)	OIR mouse model + VEGF-stimulated HUVEC angiogenic assays	Pathological retinal neovascular tufts/vascular endothelium; inner retinal glia and microglia	Nattokinase; intravitreal brusatol in OIR mice; brusatol and Nrf2 siRNA in HUVEC assays	Level 1: *in vitro* Nrf2 loss-of-function support; *in vivo* brusatol attenuated NK-associated anti-neovascular protection.	Reduced pathological angiogenic/inflammatory signaling, reactive gliosis, and pro-inflammatory microglial phenotype.	Neovascular tufts/nuclei, avascular area, and vascular leakage were reduced; VEGF-induced HUVEC angiogenic behaviors were suppressed.
(98)	Hypobaric hypoxia-induced rat retinal injury model	Whole retina; RGC and plexiform layers; photoreceptor-layer caspase-3 immunoreactivity assessed	HSD during hypobaric hypoxia	Level 3: Restoration of Nrf2/HO-1 signaling associated with protection; no reverse validation.	Restored Nrf2/HO-1 signaling and reduced intrinsic apoptosis-related changes, with PARP1 reduction and CNTF preservation.	Hypobaric hypoxia-induced TUNEL-positive retinal cells were reduced.
(99)	OIR mouse model + H₂O₂-induced RGC oxidative-stress model	Retinal tissue; GCL/RGCs; VEGF-positive retinal vasculature	Melatonin; miR-23a-3p antagomir; H₂O₂-induced RGC assay	Level 5: Direct miR-23a-3p–Nrf2 upstream evidence; no direct Nrf2/HO-1 loss-of-function validation.	Reduced oxidative stress, angiogenic/proliferative response, and retinal/RGC apoptosis with miR-23a-3p downregulation.	OIR-associated pathological changes and GCL injury were partly improved; H₂O₂-induced RGC apoptosis decreased.
(96)	Anterior-chamber IOP elevation-induced mouse retinal I/R model	RGCs/inner retina	Brazilian green propolis after retinal I/R	Level 3: Nrf2/HO-1 upregulation associated with RGC protection; no reverse validation.	Reduced HIF-1α/GFAP responses, NF-κB activation, and Bax/p53-related apoptosis signaling; preserved histone acetylation and increased Nrf2/HO-1.	Brn3a-positive RGC survival increased and ischemia-associated RGC loss decreased.
(97)	Anterior-chamber IOP elevation-induced rat retinal I/R injury model	Whole retina/retinal neural layers	NAS plus intravitreal aflibercept; NAS comparator	Level 6: Combination-related Akt/Nrf2/HO-1 association; no inhibitor/genetic validation or aflibercept-only arm.	Reduced oxidative stress and apoptosis; improved antioxidant enzyme activity.	Retinal histopathological damage, retinal thickening, and apoptosis were reduced after retinal I/R injury.
(56)	Anterior-chamber perfusion-induced rat retinal I/R model	Retinal NVU: RGC function, glia, microvasculature, and endothelial junctions	GLP after retinal I/R	Level 3: Nrf2/HO-1 upregulation associated with protection; no reverse validation.	Reduced oxidative stress, apoptosis, gliosis/microglial activation, and vascular/NVU remodeling signals.	Retinal thinning/disorganization was attenuated; ERG and PhNR amplitudes, capillary perfusion density, and vascular tortuosity improved.
Photoreceptor degeneration/photo-oxidative retinal injury						
(44)	rd10 mouse RP model	Photoreceptors/ONL; whole retinal tissue for Nrf2/HO-1 assessment	FS extract; LeDing comparator	Level 3: Nrf2/HO-1 upregulation associated with protection; no reverse validation.	Enhanced antioxidant defense and reduced lipid peroxidation.	ONL thickness/cell number and scotopic ERG a-/b-wave amplitudes improved.
(88)	RCS rat Mertk-deficient RP model + MERTK-silenced donor-derived primary human RPE	RPE/POS phagocytosis; photoreceptors/outer retina *in vivo*	Intravitreal Fer-1; MERTK, NFE2L2, or HMOX1 siRNA in HsRPE	Level 1: *in vitro* NFE2L2/HMOX1 loss-of-function evidence; silencing rescued MERTK-deficient HsRPE dysfunction.	Reduced Fe^2+^ accumulation and ferroptosis-related stress; restored cytoskeletal organization and POS uptake.	Retinal thickness and scotopic ERG responses improved in RCS rats; HsRPE phagocytosis and cytoskeletal integrity were restored.
(70)	Blue LED light-induced rat retinal injury model + blue light-exposed ARPE-19 cells	Outer retina/retinal mitochondria; ARPE-19 cells	BLM before blue-light injury; ML385 for *in vitro* Nrf2-inhibition validation	Level 2: *in vitro* pharmacological support for Nrf2 involvement; ML385 attenuated BLM-associated cytoprotection.	Reduced mitochondrial oxidative stress and senescence markers; restored Nrf2/HO-1 signaling.	Retinal histopathological changes were improved; ARPE-19 viability increased.
(43)	White LED light-induced mouse LIRD model + H₂O₂-treated 661w photoreceptor-derived cells	Photoreceptors/ONL; 661w photoreceptor-like cells	AAV2/8-mediated HMGB2 knockdown in LIRD mice; HMGB2 manipulation in H₂O₂-treated 661w cells	Level 5: Upstream HMGB2 loss-of-function evidence; no direct Nrf2/HO-1 manipulation.	Suppressed HMGB2/RAGE-NF-κB/NLRP3 inflammasome activation, pyroptosis, and photoreceptor-like cell death; restored Nrf2/HO-1 response.	ONL thinning and photoreceptor loss were attenuated; scotopic ERG a-/b-wave amplitudes were partly preserved; H₂O₂-induced 661w cell death decreased.
(76)	LED-induced rat photo-oxidative retinal damage model	Photoreceptors/ONL; whole retinal tissue	L/Z, QC, QCG, or L/Z + QCG before LED exposure	Level 6: Nutritional combination-related Nrf2/HO-1 association; no pathway blockade/genetic validation.	Reduced oxidative/inflammatory stress; restored antioxidant activity and neuroplasticity-related markers.	ONL thickness was preserved and LED-induced retinal structural damage was attenuated.

Nrf2/HO-1-related evidence levels follow the pathway-specific grading framework described in the main text and do not indicate overall therapeutic efficacy. Pharmacological inhibitor-based evidence was interpreted cautiously when inhibitor specificity was uncertain.

## 6 Therapeutic strategies involving Nrf2/HO-1 signaling

### 6.1 Natural products and phytochemicals

Naturally derived bioactive compounds represent a major class of preclinical strategies associated with Nrf2/HO-1-related retinal protection. In DR-related models, R-Rg3 showed protective effects in HFD/STZ-induced T2DM mouse retinas and HG-treated HRECs (54), whereas RSV improved retinal morphology and electrophysiological responses and reduced RGC apoptosis in STZ-induced diabetic mice and HG-treated primary RGCs (35). In HRECs, ML385 weakened R-Rg3-associated endothelial protection, providing *in vitro* pharmacological support for Nrf2 involvement (54), whereas in HG-treated primary RGCs, Nrf2 siRNA attenuated RSV-associated cytoprotection, providing *in vitro* genetic loss-of-function evidence for partial Nrf2 dependence in this cellular model (35).

In glaucoma-related RGC injury, astaxanthin and nobiletin attenuated RGC apoptosis, oxidative stress, and retinal injury; Nrf2 deficiency or HO-1 inhibition weakened their protective effects, supporting Nrf2/HO-1 involvement in these natural-product-associated interventions (39, 40).

In RPE/dry AMD-like injury, glycyrrhizin represents a natural-product candidate associated with Akt–Nrf2/HO-1-related signaling and protection against sodium iodate-induced retinal degeneration (91). As a natural polysaccharide-based intervention, GLP produced preclinical NVU-level protection in RIR rats, with Nrf2/HO-1 upregulation accompanying reduced oxidative/glial injury and improved functional and vascular outcomes (56).

### 6.2 Drug repurposing and synthetic small molecules

Repurposed drugs and synthetic small molecules represent non-natural-product strategies for Nrf2/HO-1-related retinal intervention, although current evidence remains largely preclinical. In chronic OHT rats, the Nrf2 activator tBHQ enhanced retinal Nrf2/HO-1 expression and was accompanied by reduced RGC apoptosis and increased RGC numbers (36). DMF increased TUBB3-positive RGC density and improved pSTR amplitudes in an ONC model, accompanied by enhanced Nrf2/HO-1 signaling in RGCs (33). Together, these findings support an association between pharmacological Nrf2/HO-1 activation and structural or functional RGC protection.

Designed Nrf2 activators further extend this strategy to retinal vascular pathology. Bardoxolone methyl and RS9 reduced VEGF-induced endothelial cell migration, whereas RS9 additionally inhibited pathological neovascularization in OIR models and reduced vascular leakage in a monkey CNV model, supporting the relevance of synthetic Nrf2 activation to pathological angiogenesis and vascular hyperpermeability (55).

Drug-targetable upstream nodes may also modulate Nrf2/HO-1-related responses indirectly. As a SIRT6 activator, MDL800 was associated with MAPK suppression, AMPK–Nrf2–HO-1/NQO1 activation, reduced microglial activation, and decreased RGC apoptosis in glaucoma-related models (60).

### 6.3 Gene/RNA-based modulation and advanced delivery platforms

#### 6.3.1 Gene/RNA-based modulation

Gene- and RNA-level modulation provides a mechanistically distinct route for Nrf2/HO-1-related intervention, but current evidence remains mainly limited to ARPE-19 cell models. In HG-treated ARPE-19 cells, FTO overexpression enhanced Nrf2/HO-1 expression and Nrf2 nuclear localization by reducing m6A modification of Nrf2 mRNA; Nrf2 or HO-1 knockdown weakened FTO-associated protection (50). Lentivirus-mediated HO-1 overexpression also reduced oxidative and apoptotic injury in H2O2-injured ARPE-19 cells (49). Together, these studies provide complementary *in vitro* genetic evidence: loss-of-function data support functional dependence of FTO-associated protection on Nrf2 and HO-1 in the HG-treated ARPE-19 model, whereas HO-1 overexpression provides gain-of-function support for cytoprotection.

#### 6.3.2 Extracellular vesicle- and nanocarrier-based delivery platforms

Extracellular vesicles (EVs) and nanocarriers provide platforms for intercellular communication and drug delivery in Nrf2/HO-1-related strategies, potentially improving retinal accessibility, local retention, or sustained release of candidate interventions. ASC-EVs attenuated H_2_O_2_-induced oxidative stress and apoptosis in ARPE-19 cells and upregulated NFE2L2/HMOX1/NQO1; NFE2L2 siRNA weakened this cytoprotection. In Royal College of Surgeons (RCS) rats, intravitreal ASC-EVs transiently preserved ONL/photoreceptor structure and ERG responses, accompanied by increased nuclear Nrf2, HO-1, and NQO1, although durability and miRNA-cargo causality remain unresolved (100). As a nanocarrier strategy, tFNAs-QUE delivered quercetin using tetrahedral framework nucleic acids and produced combined anti-angiogenic and neuroprotective effects in hypoxic HUVECs and OIR models, accompanied by Akt/Nrf2/HO-1-related signaling activation (87). As a nanodelivery strategy, mPEG-PLGA encapsulation prolonged GPA release and improved ocular delivery properties, producing stronger retinal microvascular protection than free GPA, accompanied by enhanced Nrf2/HO-1/GPX4-related anti-ferroptotic responses (13). Together, these studies support EV- and nanocarrier-based delivery as feasible approaches for Nrf2/HO-1-related retinal protection, although retinal cell-type specificity, delivery durability, and causal pathway validation remain insufficiently defined. For EV-based approaches, further translational barriers include standardization of EV isolation and characterization, batch-to-batch consistency, and attribution of protective effects to defined Nrf2/HO-1-modulating cargo (101).

### 6.4 Long-acting and combination strategies

Beyond individual agents and delivery platforms, long-acting and combination strategies have been explored to improve treatment durability or broaden therapeutic coverage. In a rat ONC model used to study TON, a single intravitreal injection of long-acting PEG-G-CSF improved fVEP responses and increased RGC survival, reduced RGC apoptosis and ED1-positive cell accumulation at the optic nerve lesion, and increased p-Akt1, Nrf2, SIRT3, and HO-1 expression in optic nerve tissue without significantly increasing peripheral leukocyte counts. These findings provide preclinical support for single-dose intraocular delivery of long-acting PEG-G-CSF, accompanied by increased Nrf2 and HO-1 expression (90).

Combination approaches have also been evaluated. NAS combined with aflibercept attenuated retinal histological injury and thickening in RIR rats, reduced apoptosis and oxidative stress, and enhanced Akt/Nrf2/HO-1-related signaling; however, the contribution of aflibercept could not be isolated in the absence of an aflibercept-only arm (97). As evidence for nutritional combination strategies, L/Z combined with QCG attenuated LED-induced photo-oxidative retinal damage, with a 20 mg/kg 1:1 L/Z + QCG regimen showing relatively favorable effects on Nrf2/HO-1 expression, inflammatory/glial activation, ONL preservation, and neuroplasticity-related markers (76). Collectively, long-acting delivery and pharmacological or nutritional combination strategies warrant further preclinical evaluation, particularly with respect to durability, component-specific effects, disease-stage applicability, and causal pathway dependence.

### 6.5 Translational barriers across therapeutic modalities

The translational value of Nrf2/HO-1-related strategies depends not only on the active intervention but also on retinal delivery, tissue exposure, formulation quality, and long-term safety. Achieving therapeutically relevant exposure in the intended retinal compartment requires an appropriate administration route and adequate access across ocular barriers, including the blood–retinal barrier, while accounting for tissue distribution, retention, and clearance (102, 103). Increased retinal exposure must be balanced against local ocular toxicity, inflammatory or immune responses, and the burden of repeated or invasive administration (102, 103). Formulation quality and product standardization, including chemical or cargo consistency, stability, particle or vesicle properties, storage conditions, and reproducibility, remain particular challenges for sustained-release, gene-based, EV-mediated, and nanocarrier-based strategies (101–103). For Nrf2/HO-1-related interventions, these considerations are further linked to defining an appropriate magnitude and duration of pathway modulation. Accordingly, translational development requires coordinated optimization of retinal targeting, exposure profile, formulation quality, therapeutic window, and long-term safety.

Using the same pathway-specific evidence-level framework described in Section 5, Table 2 summarizes representative therapeutic strategies involving Nrf2/HO-1 signaling, together with their evidence levels and key translational limitations.

**Table 2:** Representative preclinical therapeutic strategies targeting or associated with Nrf2/HO-1 signaling and key translational limitations in RNDs.

Ref.	Representative intervention	Disease/model context	Main protective effects	Nrf2/HO-1 evidence level	Key translational limitations
Natural products and phytochemicals					
(54)	R-Rg3	HFD/STZ-induced T2DM mouse diabetic retinal injury; HG-induced HREC injury	Reduced diabetic retinal microvascular injury and endothelial barrier dysfunction.	Level 2: *in vitro* pharmacological support for Nrf2 involvement; ML385 attenuated R-Rg3-associated endothelial protection.	*In vivo* pathway dependence, retinal exposure, dose window, and long-term safety remain unresolved.
(39)	Astaxanthin/AST, oral gavage	High-IOP-induced retinal ischemia–reperfusion mouse model; WT and Nrf2−/− mice	Preserved retinal structure and RGC survival after acute I/R injury.	Level 1: *in vivo* Nrf2 knockout evidence; Nrf2 deficiency attenuated AST-mediated retinal/RGC protection.	Acute I/R model and peri-injury oral dosing; HO-1-specific causality, durability and long-term safety remain unresolved.
(91)	Glycyrrhizin	Sodium iodate-induced ARPE-19 oxidative/apoptotic injury and mouse retinal/RPE injury; atrophic AMD-like injury model.	Attenuated RPE/outer retinal injury in an atrophic AMD-like model.	Level 5: Akt–Nrf2/HO-1 upstream regulator-associated evidence; no direct Nrf2/HO-1 loss-of-function validation.	Nrf2/HO-1 causality untested; AKT inhibition limited to ARPE-19 signaling; SI model is AMD-like toxic injury.
(40)	Nobiletin/NOB, intravitreal administration	Microbead-induced ocular hypertension rat model; hypoxia-treated primary RGCs	Improved RGC survival, inner retinal preservation, and PhNR responses.	Level 2: *in vivo* pharmacological support for HO-1 involvement; SnPP attenuated NOB-associated RGC protection.	Preclinical OHT/hypoxia only; repeated prophylactic intravitreal dosing, SnPP specificity, Nrf2 causality, and long-term safety remain unresolved.
(35)	RSV	STZ-induced diabetic mouse model; high-glucose-treated primary RGCs	Improved diabetic retinal morphology and RGC-related functional outcomes.	Level 1: *in vitro* Nrf2 loss-of-function evidence; Nrf2 siRNA attenuated RSV-associated protection in HG-treated primary RGCs.	Nrf2 dependence was validated only in cultured RGCs; *in vivo* genetic validation, bioavailability, and chronic safety remain unresolved.
(56)	Ganoderma lucidum polysaccharides/GLP, daily intragastric administration	Anterior chamber perfusion-induced retinal ischemia–reperfusion rat model	Preserved retinal structure/function and attenuated NVU-related impairment.	Level 3: Nrf2/HO-1 upregulation associated with protection; no reverse validation.	Animal-only evidence; Nrf2/HO-1 dependence, retinal exposure/BRB penetration, and polysaccharide standardization remain unresolved.
Drug repurposing and synthetic small molecules					
(36)	tBHQ, oral Nrf2 activator	Rat chronic ocular hypertension model of glaucoma	Improved RGC preservation in a chronic ocular hypertension model.	Level 3: Pharmacological Nrf2 activation associated with RGC protection; no reverse pathway validation.	Single COH rat model; no reverse pathway validation or functional electrophysiology; retinal exposure, tBHQ specificity, and long-term safety remain unresolved.
(33)	Dimethyl fumarate/DMF, oral administration	Mouse optic nerve crush model; RGC-focused retinal analysis	Enhanced RGC survival and partially preserved pSTR responses after ONC.	Level 3: Nrf2 nuclear accumulation/HO-1 upregulation associated with RGC protection; no loss-of-function validation.	Acute ONC model with a 7-day endpoint; retinal exposure and Nrf2/HO-1 dependence remain unresolved.
(55)	RS9; bardoxolone methyl/BARD	VEGF-treated HRMECs; mouse OIR model; laser-induced monkey CNV angiogenic/hyperpermeability model	Reduced endothelial hyperpermeability, OIR neovascularization, and CNV leakage.	Level 3: Pharmacological Nrf2 activation associated with vascular protection; no pathway-specific loss-of-function validation.	Cross-model preclinical evidence; no significant reduction in monkey CNV lesion size, and pathway dependence, ocular PK/durability, and disease specificity remain unresolved.
(60)	MDL800	LPS-induced BV2 microglial inflammation; BV2–R28 retinal precursor co-culture; acute OHT/ischemia–reperfusion-like rat model	Reduced microglia-associated inflammatory injury and RGC-related damage.	Level 5: Upstream SIRT6 loss-of-function support; SIRT6 inhibition/knockdown attenuated MDL800 responses without direct Nrf2/HO-1 manipulation.	BV2/R28 surrogate and short-term acute OHT models; Nrf2/HO-1-specific dependence, microglia-specific targeting, and long-term safety remain unresolved.
Gene/RNA-based modulation					
(50)	FTO overexpression/m6A demethylase modulation	High-glucose-treated ARPE-19 cells; DR-related RPE oxidative injury model *in vitro*	Improved ARPE-19 redox balance and viability under high-glucose stress.	Level 1: *in vitro* Nrf2/HO-1 loss-of-function evidence; Nrf2 or HO-1 knockdown attenuated FTO-associated cytoprotection.	*In vitro* ARPE-19 evidence only; no *in vivo* FTO functional validation; retinal delivery, target specificity and long-term epitranscriptomic effects remain unresolved.
(49)	Lentiviral HO-1 overexpression	H₂O₂-induced ARPE-19 oxidative/apoptotic injury model	Reduced oxidative/apoptotic injury and preserved mitochondrial integrity in ARPE-19 cells.	Level 1: *in vitro* HO-1 genetic gain-of-function evidence; HO-1 overexpression improved ARPE-19 cytoprotection.	*In vitro* ARPE-19 only; endogenous HO-1 necessity and Nrf2 causality were not tested, and *in vivo* viral delivery safety/expression control remain unresolved.
EV/nanocarrier-based delivery					
(87)	tFNAs-QUE	Hypoxia-treated HUVECs; mouse OIR model with intravitreal tFNAs-QUE	Reduced OIR-related angiogenic injury and preserved retinal neuronal structure and ERG responses.	Level 6: Nanodelivery-related AKT/Nrf2/HO-1 association; no pathway-specific loss-of-function validation.	HUVEC/OIR preclinical evidence; pathway dependence, retinal biodistribution, long-term material safety, dosing durability, and reproducibility remain unresolved.
(13)	Topical ocular GPA-loaded mPEG-PLGA nanoparticles/GPA-NP	db/db diabetic mouse retinal microvasculopathy; high-glucose-treated HRMECs	Reduced diabetic retinal vascular leakage and endothelial ferroptotic injury.	Level 5: Direct Keap1-targeting/upstream evidence; Nrf2/HO-1/GPX4 upregulation occurred without direct Nrf2/HO-1 loss-of-function validation.	Preclinical DR nanodelivery only; Nrf2/HO-1 dependence remains untested, posterior-segment exposure remains limited, and long-term dosing and formulation reproducibility require further validation.
(100)	ASC-EVs	H₂O₂-treated ARPE-19 cells; RCS retinal degeneration rats receiving intravitreal ASC-EVs	Reduced ARPE-19 oxidative injury and transiently preserved photoreceptor/ERG outcomes *in vivo*.	Level 1: *in vitro* Nrf2 loss-of-function evidence; NFE2L2 siRNA attenuated ASC-EV cytoprotection, whereas *in vivo* pathway activation remained associative.	Transient *in vivo* efficacy; *in vivo* retinal Nrf2 dependence, miRNA cargo causality, RPE-specific effects, EV consistency, and long-term safety remain unresolved.
Delivery-optimized and combination strategies					
(76)	Lutein/zeaxanthin isomers + quercetagetin/L/Z + QCG	LED-induced photo-oxidative retinal damage in rats	Preserved ONL structure and attenuated photo-oxidative retinal injury.	Level 6: Nutritional combination-related Nrf2/HO-1 association; no pathway-specific reverse validation.	LED photo-oxidative injury model only; pathway dependence, clinically relevant dose/ratio and duration, and generalizability to human retinal degeneration remain unresolved.
(90)	Intravitreal PEG-G-CSF	Rat optic nerve crush/TON-related model with intravitreal PEG-G-CSF	Preserved FVEP responses and RGC density after optic nerve injury.	Level 6: Long-acting delivery-related optic-nerve Nrf2/HO-1 association; no pathway-specific reverse validation.	Preclinical ONC model only; nuclear Nrf2 activation, pathway dependence, durability beyond the short follow-up, repeat dosing, and long-term ocular safety remain unresolved.
(97)	N-acetylserotonin + aflibercept/NAS + aflibercept	Elevated IOP-induced retinal ischemia–reperfusion injury rat model	Attenuated retinal structural injury, apoptosis, and oxidative stress after RIR.	Level 6: Combination-related Akt/Nrf2/HO-1 association; no pathway-specific reverse validation or aflibercept-only arm.	Animal RIR model only; pathway dependence and the component-specific contribution or synergy of aflibercept cannot be determined.

The pathway-specific grading framework described in the main text was applied to the therapeutic strategies summarized in this table. Evidence levels do not indicate overall therapeutic efficacy or translational readiness. Pharmacological inhibitor-based evidence was interpreted cautiously when inhibitor specificity was uncertain.

## 7 Limitations and evidence gaps

Although Nrf2/HO-1 is repeatedly implicated in RND-related stress responses, it should not be generalized as a uniformly protective therapeutic target. Across RND models, retinal cell types, injury paradigms, and intervention strategies, much of the available evidence still reflects parallel changes between pathway modulation and protective phenotypes rather than definitive causal dependence. Therefore, the causal role and context dependence of this axis require further clarification.

Another limitation is that the evidence base remains predominantly preclinical. As summarized in Tables 1, 2, most studies reviewed here used cultured retinal cells or rodent models. Although these models are useful for dissecting pathway-associated stress responses, they do not fully recapitulate human retinal architecture, macular specialization, vascular organization, chronic disease progression, or the immune–glial microenvironment. Human-relevant evidence is limited to a small number of studies using patient-derived iPSC-RPE, donor-derived primary RPE, or retinal organoids, and causal validation in these systems remains incomplete. Accordingly, the current evidence does not yet permit conclusions regarding clinical efficacy or safety.

Substantial heterogeneity also exists across disease contexts and experimental designs. Disease subtypes and stages, such as dry versus neovascular AMD, non-proliferative versus proliferative DR, and acute versus chronic glaucoma-related injury, represent distinct pathological settings rather than interchangeable RND contexts. Across these settings, and even among studies using similar interventions or disease models, the timing, magnitude, cellular localization, and kinetics of Nrf2/HO-1 activation may differ, while intervention route, dose, therapeutic exposure, treatment window, observation period, and endpoint selection vary widely. Such heterogeneity limits direct cross-study comparison and makes it difficult to define a unified therapeutic window for Nrf2/HO-1 modulation across RNDs.

Finally, adverse or context-dependent outcomes remain incompletely characterized. Potentially maladaptive contexts, including HO-1-related labile iron release, ferroptosis-prone RPE injury, angiogenic retinal microenvironments, and parallel activation of Nrf2/HO-1 and pro-inflammatory cascades, require more careful mechanistic validation. The evidence grading framework used in this review is qualitative and pathway-focused; it is intended to organize the strength of Nrf2/HO-1-related support rather than to serve as a formal risk-of-bias assessment or quantitative evidence synthesis. Therefore, the evidence levels should be interpreted as an organizing framework for mechanistic and preclinical findings rather than as definitive indicators of therapeutic readiness.

## 8 Conclusions and future perspectives

In summary, the Nrf2/HO-1 signaling axis represents an important redox-adaptive pathway that links oxidative stress defense, cell-injury control, and retinal homeostasis in RNDs. Current preclinical evidence shows that enhancement of Nrf2/HO-1-related signaling is frequently accompanied by reduced oxidative burden, alleviated mitochondrial injury, attenuation of inflammatory and cell-death responses, and improvement of retinal structure or function, suggesting that this axis may constitute an important, context-sensitive stress-response node within the pathological stress network of RNDs.

Future studies should move beyond measuring Nrf2 or HO-1 expression and establish whether pathway modulation is causally required for protection in defined retinal cell types, disease subtypes, and stages. Cell type-specific genetic manipulation, temporal pathway profiling, and structural and functional outcome assessment should be combined to distinguish adaptive compensation from mechanistic involvement or protective dependence. Single-cell and spatial approaches may further clarify how Nrf2/HO-1 responses vary across major retinal neuronal, epithelial, vascular, and glial cell populations, and how these responses change during acute injury, chronic degeneration, or angiogenic remodeling.

Future human-relevant validation should build on existing iPSC-RPE, primary RPE, and retinal organoid studies through direct NFE2L2/HMOX1 perturbation and broader patient representation, while extending validation to donor retinal tissue and neurovascular or immune–glial multicellular models. Cross-species comparisons should determine whether pathway activation, cellular localization, treatment response, and toxicity observed in rodents are conserved in human retinal compartments before candidate interventions advance toward clinical evaluation.

From a translational perspective, development should focus not only on identifying additional Nrf2/HO-1 modulators, but also on defining the disease context, target retinal compartment, magnitude and duration of modulation, delivery route, and treatment window most likely to produce a net protective effect. Candidates should be prioritized for further development based on causal pathway evidence, target-cell specificity, reproducible structural and functional benefit, feasible delivery, and an acceptable safety window. Once clinical evaluation is warranted, studies should incorporate prespecified pharmacodynamic or redox-related biomarkers, where feasible, together with standardized imaging and visual-function outcomes, while monitoring local ocular toxicity and potential adverse effects of sustained or excessive pathway modulation.
